# Editorial: Biological Mechanism-Based and Patient-Centered Management of Cancer-Related Symptoms and Syndromes

**DOI:** 10.3389/fphys.2018.01819

**Published:** 2018-12-18

**Authors:** Antonio Macciò, Silvia Busquets, Clelia Madeddu, Josep M. Argilés

**Affiliations:** ^1^Department of Gynecologic Oncology, Azienda Ospedaliera Brotzu, Cagliari, Italy; ^2^Cancer Research Group, Department de Bioquimica i Biomedicina Molecular, Facultat de Biologia, Universidad de Barcelona, Barcelona, Spain; ^3^Institut de Biomedicina de la Universitat de Barcelona, Barcelona, Spain; ^4^Department of Medical Sciences and Public Healt, University of Cagliarih, Cagliari, Italy

**Keywords:** cancer cachexia, anorexia, muscle wasting, quality of life, supportive care, inflammation, interleukin 6, cancer-related anemia

In recent years, full recovery rates for cancer patients significantly increased and mean survival improved. Moreover, chronicization of cancer disease and concerns about aggressive care close to the end-of-life raised the awareness of better risk-benefit balancing. As a result, patients are increasingly aware of quality of life (QoL) issues, and physicians need to find the right balance between cancer treatment and patient well-being. Notably, for most tumors, especially at advanced stages, the concept of cancer as a multidimensional systemic disease replaced the idea of cancer as an organ-confined disease. A new way of design of antineoplastic treatments aiming to patient-centered outcomes including clinical benefit, symptom control, and psycho-social well-being is needed. For this scope, the notion of simultaneous care, i.e., early integration of palliative care into the cancer disease trajectory, must be pursued in clinical practice.

Cancer-associated symptoms, such as anorexia, fatigue, depression, and syndromes such as cachexia, appear more frequently in advanced stages but they actually start with the onset of cancer. Therefore, any antineoplastic therapeutic approach should ideally be planned within the context of “best supportive care,” including optimal symptom management and careful psychosocial and spiritual counseling (Madeddu et al., 2015). However, to date the exact biological mechanisms of cancer-associated symptoms and their specific treatment have not been well defined yet.

This Research Topic aimed to gain the knowledge of the pathophysiology of cancer symptoms and syndromes to implement a proactive structured evaluation and targeted interventions. We included just some significant conditions, namely anorexia, weight loss/cachexia, and anemia that are very important for patient suffering, although yet unmeet concerns in clinical practice. The final aim is to develop a model, which could be applied also to other symptoms or syndromes.

This Topic provides significant contributions in the issue of cancer-related muscle wasting and cachexia pathogenesis and treatment. Interestingly, Barreto et al. analyzed the proteomic signature of muscle wasting induced by different conditions (i.e., cancer cachexia and chemotherapy). They showed a significant activation mainly of the pathways that regulate nucleotide and fatty acid metabolism, ATP synthesis, muscle and heart function, and ROS scavenging. This evidence has important translational implications and supports a combination strategy as the potentially most effective treatment for cachexia. This evidence is further supported by Pin et al. which suggested that inhibiting a single proteolytic pathway is not a good strategy to contrast cancer-induced muscle wasting. In fact, they showed that the inhibition of the Ca^2+^-dependent proteases did not change body weight loss and muscle wasting in some animal experimental models of cancer cachexia.

As a consequence of changes in body composition and energy metabolism, as well as of treatment-related toxicities, advanced cancer patients very often show a decreased tolerance to exercise and reduced levels of physical activity. Marmonti et al. present a model of bed-rest induced muscle wasting, demonstrating how inactivity may contribute to muscle atrophy associated with a significant decline in muscle mass and force, as well as bone mass loss. Similarly, Bonetto et al. demonstrated that bone loss is associated with tumor growth in two experimental models of colorectal cancer cachexia (HT-29, and Apc^Min/+^).

A fundamental step in establishing an effective targeted supportive care of cancer symptoms is represented by their structured multidimensional evaluation.  Argilés et al. presents the validation of the new tool Cachexia Score (CASCO) and its shorten version miniCASCO, for the diagnosis and numerical staging of cancer cachexia in different level of severity.

One of the most frequent and clinically relevant signs of advanced stage cancer patients is cancer-related anemia (CRA). The review by Madeddu et al. is focused on the main and novel mechanisms involved in the multifactorial pathogenesis of CRA and provides useful insights for the development of an effective mechanism-based approach, which may be able to promote a relevant amelioration of patients' quality of life.

During the last years, it has become very clear that a combination of nutrition, nutraceuticals, and drugs is a much-preferred therapeutic approach to fight against cancer cachexia. In the present Research Topic different therapeutic approaches have been proposed. Blauwhoff-Buskermolen et al. showed in a population of advanced non-small-cell lung cancer patients that those with anorexia (one of the main features of cachexia) had significantly higher ghrelin levels compared to those without anorexia. Indeed, ghrelin plays an important orexigenic role by stimulating the production of orexigenic neurons such as neuropeptide Y. Then, anorectic/cachectic cancer patients can benefit from a treatment with a ghrelin receptor agonist (Garcia et al., 2013; Temel et al., 2016). Interleukin-6 (IL-6) is the main proinflammatory cytokines involved in the etiopathogenenesis of the main symptoms and syndromes of advanced cancer, including CRA (Macciò et al., 2005) and cachexia (Zimmers et al., 2016). Consistently, IL-6 inhibitors obtained promising results in the treatment of cachexia and related symptoms (Bayliss et al., 2011; Bekaii-Saab et al., 2011; Prado et al., 2012). In our topic, Au et al. investigated the potential mechanisms of Selumetinib (mitogen-activated protein/extracellular signal-regulated kinase (MEK) inhibitor) in Lewis lung carcinoma, an experimental animal model of cancer cachexia. Selumetinib reduced tumor mass and circulating and tumor IL-6, but did not preserve muscle mass. Another strategy is discussed by Molfino et al. they showed that a dietary supplementation with docosahexaenoic acid (DHA) was associated with increased DHA levels and omega-3 index in red blood cells membranes of breast cancer patients.

We hope that the present Research Topic could contribute a greater insight to enable earlier and more effective supportive and palliative care for cancer patients. In fact, although traditional cornerstones, such as diagnosis, staging, and anticancer treatment will continue to play a crucial role, the knowledge of the biological specificity of cancer-associated symptoms should be considered a central issue by the multidisciplinary team to optimize a patient-focused care. We are awareness that such multidimensional personalized approach should include also the assessment of the psychosocial and spiritual domains, which can help patients to develop personal priorities regarding relationships, religious and spiritual beliefs, deal with the urgency of resolving conflicts, and achieve personally meaningful goals.

## Author Contributions

All authors listed have made a substantial, direct and intellectual contribution to the work, and approved it for publication.

### Conflict of Interest Statement

The authors declare that the research was conducted in the absence of any commercial or financial relationships that could be construed as a potential conflict of interest.

## References

[B1] Bayliss T. J. Smith J. T. Schuster M. Dragnev K. H. Rigas J. R. (2011). A humanized anti-IL-6 antibody (ALD518) in non-small cell lung cancer. Expert Opin. Biol. Ther. 11, 1663–1668. 10.1517/14712598.2011.627850 21995322

[B2] Bekaii-Saab T. Phelps M. A. Li X. Saji M. Goff L. Kauh J. S.. (2011). Multi-institutional phase II study of selumetinib in patients with metastatic biliary cancers. J. Clin. Oncol. 29, 2357–2363. 10.1200/JCO.2010.33.9473 21519026 PMC3107751

[B3] Garcia J. M. Friend J. Allen S. (2013). Therapeutic potential of anamorelin, a novel, oral ghrelin mimetic, in patients with cancer-related cachexia: a multicenter, randomized, double-blind, crossover, pilot study. Support Care Cancer 21, 129–137. 10.1007/s00520-012-1500-1 22699302

[B4] Macciò A. Madeddu C. Massa D. Mudu M. C. Lusso M. R. Gramignano G.. (2005). Hemoglobin levels correlate with interleukin-6 levels in patients with advanced untreated epithelial ovarian cancer: role of inflammation in cancer-related anemia. Blood 106, 362–367. 10.1182/blood-2005-01-0160 15774616

[B5] Madeddu C. Mantovani G. Gramignano G. Macci,ò A. (2015). Advances in pharmacologic strategies for cancer cachexia. Expert Opin. Pharmacother. 16, 2163–2177. 10.1517/14656566.2015.1079621. 26330024

[B6] Prado C. M. Bekaii-Saab T. Doyle L. A. Shrestha S. Ghosh S. Baracos V. E.. (2012). Skeletal muscle anabolism is a side effect of therapy with the MEK inhibitor: selumetinib in patients with cholangiocarcinoma. Br. J. Cancer 106, 1583–1586. 10.1038/bjc.2012.144 22510747 PMC3349178

[B7] Temel J. S. Abernethy A. P. Currow D. C. Friend J. Duus E. M. Yan Y.. (2016). Anamorelin in patients with non-small-cell lung cancer and cachexia (ROMANA 1 and ROMANA 2): results from two randomised, double-blind, phase 3 trials. Lancet Oncol. 17, 519–531. 10.1016/S1470-2045(15)00558-6 26906526

[B8] Zimmers T. A. Fishel M. L. Bonetto A. (2016). STAT3 in the systemic inflammation of cancer cachexia. Semin. Cell Dev. Biol. 54, 28–41. 10.1016/j.semcdb.2016.02.009 26860754 PMC4867234

